# Assessment of Thermosonication as Postharvest Treatment Applied on Whole Tomato Fruits: Optimization and Validation †

**DOI:** 10.3390/foods8120649

**Published:** 2019-12-06

**Authors:** Joaquina Pinheiro, Rui Ganhão, Elsa M. Gonçalves, Cristina L.M. Silva

**Affiliations:** 1 MARE—Marine and Environmental Sciences Centre, Instituto Politécnico de Leiria, 2520-630 Peniche, Portugal; rganhao@ipleiria.pt; 2 Escola Superior de Turismo e Tecnologia do Mar, Instituto Politécnico de Leiria, 2520-641 Peniche, Portugal; 3 UTI—Unidade de Tecnologia e Inovação, Instituto Nacional de Investigação Veterinária e Agrária, Avenida da República, Quinta do Marquês, 2780-157 Oeiras, Portugal; elsa.goncalves@iniav.pt; 4 GeoBiotec—GeoBioTec Research Institute, Universidade Nova de Lisboa, Campus de Caparica, 2829-516 Caparica, Portugal; 5 Universidade Católica Portuguesa, CBQF—Centro de Biotecnologia e Química Fina—Laboratório Associado, Escola Superior de Biotecnologia, Rua Diogo Botelho 1327, 4169-005 Porto, Portugal; clsilva@porto.ucp.pt

**Keywords:** tomato, thermosonication, postharvest treatment, quality, storage

## Abstract

Tomatoes are a popular and rich fruit due to their nutritional and bioactive composition as vitamins, antioxidants, and phenolics contributing to the promotion of consumer health. For this reason, emerging postharvest technologies need to be evaluated to achieve the maintenance of sensorial and quality-related characteristics, like color and texture, while aiding to fruit decontamination. Optimization of thermosonication as postharvest treatments on whole, mature-green tomatoes (cv. “Zinac”) to improve quality (color, texture, total phenolic content, and weight loss) was performed by response surface methodology. Temperature (32–48 °C), treatment time (13–47 min), and storage period at 10 °C (1–15 days) at constant ultrasound frequency (45 kHz; 80% power level), were the independent variables. In general, thermosonication delayed tomato color changes while achieving total phenolic content increase and good overall quality. Three optimal thermosonication conditions were selected and validated (32 °C-13 min, 35 °C-20 min and 40 °C-30 min). The most suitable thermosonication condition that promoted a longer storage while keeping a high-quality standard was at 40 °C during 30 min. This study demonstrated that thermosonication provides an effective alternative methodology to guarantee tomato quality without significant change during the expected postharvest period.

## 1. Introduction

The shelf-life extension of fruit is a challenge for producers, distributors, and industries, where investigation of alternative decontamination and postharvest treatments, using less severe and innovative processes, is continuously under development and subject to testing [1]. In recent years, several studies regarding the hot water treatment (HWT) effectiveness on control insect infestations, microbial growth, and delaying fruit response to extreme storage temperatures has been evaluated [2,3]. Treatment times and temperatures vary widely depending on the products to be treated and objective established. HWT promotes changes in the biochemical pathways involved in the ripening process, resulting in remarkably beneficial effects on the postharvest quality maintenance and storage life extension of fruit [3]. Several postharvest studies reported the benefits of HWT to delay softening during storage of whole fruits, like tomatoes [4], bananas “Kluai Khai” [5], and strawberry fruits [6].

Ultrasound (US) technology is a promising methodology used in fruit and vegetable decontamination and preservation [7,8,9]. The mechanism of action is based on the concept that in subjecting a liquid to ultrasonic treatment and acoustic, cavitations are created (involving formation of a multiple bubble system and bubble coalescence process) [10]. The bubbles intensely collapse when maximum size is attained, thus creating changes in the temperature and pressure [10]. The ultrasound is a non-thermal technology which contributes to the increase of microbial safety and prolongs shelf-life, especially in food with heat-sensitive, nutritional, sensory, and functional characteristics [11,12]. Cao et al. [13] studied the effects of ultrasound treatments on postharvest strawberry fruit and results showed that US at optimal conditions (250 W of ultrasonic power with treatment time of 9.8 min) delays fruit decay and microbial load compared with untreated samples. As referred by Meng et al. [14], ultrasound treatment delays the time at which the peak respiration rate occurs and lowers the magnitude of this peak respiration rate in kiwifruit.

The combination of ultrasound with some non-thermal and/or thermal methods constitutes an attractive approach to enhance microbial inactivation in fruit and vegetables [15,16]. The application of technologies such as HWT and US can also be done in combination, known as thermosonication (TS). The synergistic effect of combined treatments allows for the inactivation of enzymes at lower temperatures and has the advantage of having a minimal effect on sensorial and nutritive product quality [11]. Previous studies showed the efficiency of thermosonication as an alternative treatment to the blanching process on vitamin C retention [17] and color preservation [18] of frozen watercress, on enzymatic inactivation of pectinmethylesterase from tomato juice [19], ascorbic acid stability from juice [20], peroxidase from watercress [21], retention of bioactive compounds of watermelon juice [22], microbial reduction on orange juice [23], and apple juice [24,25]. However, little information is available regarding the use of thermosonication as a postharvest treatment to preserve whole fruit. 

Thermosonication efficiency, as a postharvest treatment, can be affected by many factors, such as the time-temperature binomial and storage period (temperature and atmosphere composition). In such situations where multiple variables may influence treatment effectiveness, the response surface methodology (RSM) approach is regarded as an effective statistical tool to aid in process optimization, with the advantage of having the ability to determine the effects of operational factors and the corresponding interactions, and modelling the response as a function of independent variables [26].

The purpose of this study was to optimize and evaluate the thermosonication as postharvest treatment for mature-green tomato fruit. Different temperatures and times at constant ultrasound frequency (45 kHz; 80% power level) and storage periods were tested. RSM was used to evaluate its effects on phenolic content and physical quality in order to extend fruit storability. Sensory attributes of the treated fruit were also evaluated.

## 2. Materials and Methods

### 2.1. Plant Material

Tomato (*Solanum lycopersicum*, cv. Zinac) fruit at mature-green stage, without physical damage and with identical characteristics in terms of color [27] and size, were purchased in a greenhouse from Carmo & Silvério in a center west of Portugal. Firstly, the fruits were briefly washed in running tap water to remove excess dirt, dried (with absorbent paper) and stored (overnight) in a cooling chamber at 10 °C until thermosonication treatments [28]. 

A physical-chemical characterization of tomato fruit without any treatment (untreated samples, Ctr control) was carried out on the initial day and after 15 days of storage at 10 °C in order to provide a baseline comparison to the thermosonication effects, and results are presented in Table 1.

### 2.2. Thermosonication Treatment

Tomato fruits (90 ± 6 g) were thermosonicated in an ultrasonic bath (Elma Transsonic multiple-frequency cleaning baths) (±1 °C) with 45 L nominal capacity at an ultrasound frequency of 45 kHz and power level of 80%, varying the time-temperature conditions according to the experimental design presented in Table 2. Treatments were carried out in baskets with ten tomatoes per treatment and immersed in water. During the treatments, the temperature of the water was monitored by means of thermocouples (type T thin thermocouple, 1.2 mm diameter, embedded in a stainless-steel hypodermic needle from Ellab, Denmark, with an accuracy of ±2 °C). After treatment, tomato fruits were dried with absorbent paper and stored at 10 °C for 15 days, as previously optimized by Pinheiro et al. [28].

### 2.3. Experimental Design

A central composite rotatable design (CCRD) was used to optimize and evaluate the main interaction and quadratic effects of the thermosonication conditions (treatment temperature: *T*, and time: *t*) and storage period (*Sp*) on tomato quality. The complete design (Table 2) consisted of three sets of experimental points: (i) a traditional factorial design with 2^k^ points, with k being the number of independent variables (factors) with coded levels +1 and –1; (ii) to account for non-linearity, a star of 2^k^ points, coded as +α and −α on the axis of the system at a distance of α = 2^k/4^ from the origin; and (iii) two central points to provide an estimate of the lack of fit of the obtained linear statistical model as well as the pure error of the experiments [29]. The independent variables taken into account were treatment time (*t*): 13–47 min; treatment temperature (*T*): 32–48 °C; and storage period (*Sp*): 1–15 days. The evaluated quality parameters (dependent variables) were color, texture, weight loss, and total phenolic content (TPC).

### 2.4. Evaluation of Optimized Thermosonication Conditions

In order to assess the effects of optimized thermosonication conditions on tomato quality and storability, fruit not subjected to any treatment (Ctr samples) and subjected to the optimal TS conditions generated by RSM were evaluated at 0, 8, and 15 days at 10 °C using the same analytical protocol considered for the experimental design and sensory analysis (color, global acceptability, and deterioration index).

### 2.5. Quality Attributes Evaluation

#### 2.5.1. Color

Color samples were evaluated according to methodology described by Pinheiro et al. [30]. Briefly, the color CIEL*a*b* parameters were obtained by colorimeter (Minolta Chroma Meter, CR-300, Osaka, Japan), after calibration using a white standard tile (L* = 97.10, a* = 0.19, b* = 1.95), and the illuminate C (10° observer). L* values represented the luminosity (0(black) to 100(white)), and a* and b* values indicated the variation of greenness to redness (−60 to +60) and blueness to yellowness (−60 to +60), respectively. Four determinations for each fruit were performed in the equatorial zone. Sixteen measurements were determined for each treatment condition.

#### 2.5.2. Texture

Texture evaluation was carried out as reported by Pinheiro et al. [30]. Briefly, the texture analyzer (TA.HDi, Stable Microsystem Ltd., Godalming, UK) with a 50 N load cell, a stainless steel cylinder probe with a 2 mm diameter, a speed of 3 mm.s^−1^, and 7.5 mm of penetration distance in the equatorial zone of the fruit was used for a penetration test. Force-distance curves were recorded and firmness (maximum peak force (N)) was used as indicator of texture. Before proceeding to texture determination of tomato samples, a period of 2 hours at room temperature was used to avoid interference of temperature. Sixteen measurements were taken for each treatment condition.

#### 2.5.3. Total Phenolic Content (TPC)

Total phenolic content (TPC) was determined using the Folin–Ciocalteau reagent as reported by Singleton & Rossi [31]. Briefly, samples (10 g) were homogenized in 70% aqueous methanol (10 mL) using a Yellow line DI 25 basic polytron (IKA-Labortechnik, Stauten, Germany), centrifuged (Sorvall RC-5, rotor SS34, DuPont, Wilmington, United States) at 43,146 g for 20 min at 4 °C and the supernatant collected. One hundred microliter of supernatant was mixed with 5 mL of Folin–Ciocalteau (1/10, *v*/*v*) and 4 mL of Na_2_CO_3_ (7.5%, *w*/*v*). The mixture was placed in a water bath (45 °C for 15 min) and the absorbance was measured at 765 nm in an ATI Unicam UV/VIS UV4 spectrophotometer (Unicom Limited, Cambridge, United Kingdom), using gallic acid as a standard. Results (six replicates) were expressed as mg of gallic acid equivalents (GAE) per kg on a fresh weight basis (mg.kg^−1^).

#### 2.5.4. Weight Loss (WL)

Weight loss (WL) was measured according to Van Dijk et al. [32]. Batches consisting of five fruit per storage temperature and day of analysis were weighted. After weighing, tomatoes were put back to original storage conditions. The weight loss was calculated relative to the weight at day zero (*t* = 0).

(1) $$\left(\%\right)\;\mathrm{Weight}\;\mathrm{loss}\;=\;\frac{W_{0}\;-\;W_{t}}{W_{0}}\;\times \;100$$

where *W*_0_ is the average weight of the first batch (three replicates) at day 0 and *W_t_* is the average weight of the same batch at day *t*.

#### 2.5.5. Sensory Analysis

Sensory analysis was performed using an analytical-descriptive test to discriminate the sensory quality attributes of Ctr and TS-treated samples along with refrigerated storage as described by Pinheiro et al. [33]. A panel of 8 trained judges (members of UEISTSA/INIAV—Unidade Estratégica de Investigação e Serviços de Tecnologia e Segurança Alimentar do Instituto Nacional de Investigação Agrária e Veterinária), who met the basic requirements of sensory sensitivity according to ISO 8586-1 (1993) [34], in adequate conditions compliant to ISO 13299 (1995) [35], identified and distinguished the sensory attributes of color, global acceptability, and deterioration index (visual evaluation) of fresh tomato fruits using the numeric rating scales as follows:

Color rating system: 1 = green (0% red); 2 = breaker (<10% red); 3 = turning (10% < red < 30%); 4 = pink (30% < red < 60%); 5 = red (60% < red < 90%) and 6 = red (>90% red).

Global acceptability rating system: 1 = highly acceptable; 2 = moderately acceptable; 3 = medium acceptable (consumer limit); 4 = moderately unacceptable; 5 = unacceptable. 

Deterioration index rating system: 0 = absent; 1 = very slight; 2 = moderate; 3 = severe. Moderate to severely deteriorated fruit are not commercialized and represent disorders affecting 25% to 50% (anchor 2) and over 50% of the fruit surface (anchor 3), respectively. 

Panelists were asked to evaluate samples during storage, scoring the level of difference in perceived intensity between TS-treated and untreated samples with respect to each attribute. 

### 2.6. Statistical Analysis

#### 2.6.1. Model Fitting and Statistical Analysis

Data were fitted to second-order polynomial equations (Equation (2)) for each dependent Y variable (L*, firmness, TPC, WL), as a function of independent variables *X_j_* (*T*, *t*, and *Sp*), through a stepwise multiple regression analysis using Statistica version 8.0 software (Tulsa, OK, USA) [36]:

(2) $$Y\;=\;b_{0}\;+\;\overset{3}{\underset{j\;=\;1}{\sum }}b_{j}X_{j}\;+\;\overset{3}{\underset{i\;<\;j}{\sum }}b_{ij}X_{i}X_{j}\;+\;\overset{3}{\underset{j\;=\;1}{\sum }}b_{jj}X_{j}^{2}$$

Where Y is the predicted response, *X_j_* is the independent variable, *b*_0_ is the intercept coefficient, *b_j_* is the linear terms, *b_jj_* is the squared terms, and *b_ij_* is the interaction terms.

The stepwise regression procedure was performed using the backward elimination method in order to remove non-significant interaction terms from the initial response surface model step by step. In each subsequent step, the least significant variable in the model was removed until all remaining variables had individual *p*-values smaller than 0.05 [37]. The criteria for eliminating a variable from the full regression equation was based on *R*^2^ values, standard error (SE) estimate, and the significance of the *F*-test and derived *p* values. The lack-of-fit and significance of the effects of each of the three independent factors was determined by analysis of variance. Three-dimensional response surface plots were generated by using plotting software [36].

To verify the accuracy of the predictive equations for the color, texture, and TPC, a total of 10 randomly selected treatment experiments, within the experimental range conditions, were replicated.

#### 2.6.2. Quality Assessment of Untreated Samples and TS-Treated at Optimal Treatment Conditions

Data from untreated (Ctr) and optimum TS-treated conditions, such as mean and standard deviation (SD) of multiple measurements, was subjected to analysis of variance [36] at *p* < 0.05 with mean separation by Tukey’s Honestly Significant Difference (HSD) test to determine thermosonication effects on tomato quality attributes for different storage days. 

## 3. Results and Discussion

### 3.1. Model Fitting

Table 3 presents the ANOVA analysis of L* color parameter, texture, TPC, and weight loss, and the corresponding predictive model equations (Equations (3)–(6)) are shown below.

(3) $$\begin{array}{ll} L*\;=\; & 73.81\;-\;0.015\;\times \;T^{2}-1.61\;\times \;t-0.070\times Sp^{2}+0.040\times T\;\times \;t+0.022\\ & \times T\;\times \;Sp \end{array}$$

(4) $$\mathrm{Firmness}\;=\;14.89-0.0013\;\times \;t^{2}-0.006\;\times \;t\;\times \;Sp$$

(5) TPC = 264.71−0.030 × T×t+0.080 × T × Sp

(6) WL = −4.96+0.15 × t + 0.023 × T × Sp−0.022 × t × Sp

The ANOVA analysis of regression models indicate that all models were significant (*p* < 0.05). The coefficients of determination (*R*^2^ and *R*^2^_adj_) explained the variability of experimental data and were satisfactory (*R*^2^ = 0.79, *R*^2^_adj_ = 0.69 for L*; *R*^2^ = 0.77, *R*^2^_adj_ = 0.71 for WL), despite the low values for firmness (*R*^2^ = 0.51, *R*^2^_adj_ = 0.43) and TPC (*R*^2^ = 0.61, *R*^2^_adj_ = 0.55). The F-test for the effectiveness of weight loss revealed that the source of variance included in the residuals is due to the inadequacy of the polynomial models to reproduce experimental data. The F-test for the lack-of-fit (Table 3) confirmed that the source of variance contained in the residuals cannot be explained by the experimental error. According to these criteria, all models were accepted for prediction, with the WL exception presenting a trend and not a predictive nature.

In order to verify the model prediction, ten sets of treatments were randomly selected from the represented contour plot and tested to validate the developed predictive model, i.e., Equations (3)–(5). The experimentally observed values were highly correlated (*R*^2^ = 0.99, 0.96, and 0.88) with the predicted values derived from Equations (3)–(5) (Table 4).

### 3.2. Response Surface Analysis

#### 3.2.1. Effect of Thermosonication on Tomato Color 

**Figure 1:**
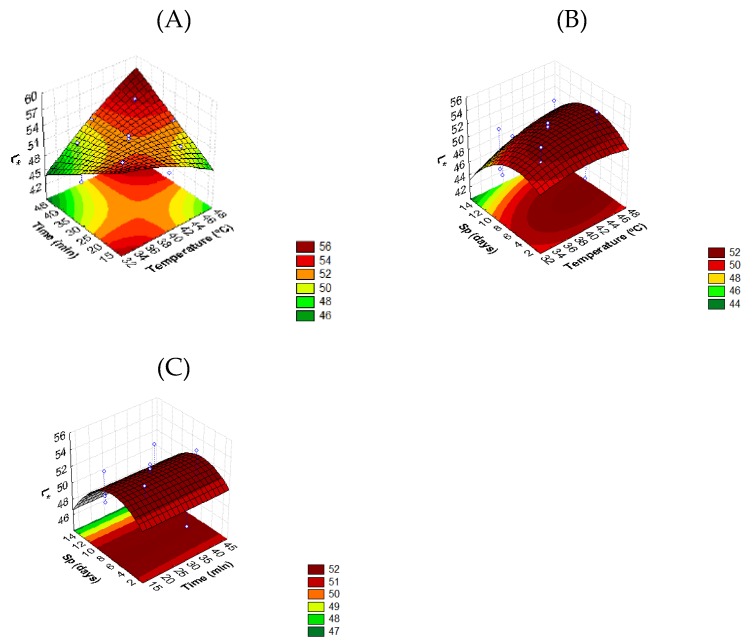
Response surface 3D contour plot indicating the effects of (**A**) temperature (*T*, °C) and time (*t*, min), (**B**) temperature (*T*, °C) and storage period (*Sp*, days), and (**C**) time (*t*, min) and storage period (*Sp*, days) on L* value of tomato fruit.

The analysis of response surface shows that both temperature (*T*) and time (*t*) of thermosonication, as well as storage period (*Sp*) have significant effects (*p* < 0.05) on whole tomato color (Figure 1).

Regarding surface models, the tomato L* value of TS-treated samples was preserved for treatment conditions in the temperature range of 30–46 °C and the time period from 10 to 35 min (Figure 1A). Moreover, thermosonication at low temperature and high treatment time (30 °C during 50 min) led to a decrease of luminosity which is not desirable since a similar value of ca. 43.8 was only recorded on Ctr samples after 15 days (Table 1).

Figure 1 presents a similar behavior of tomato L* color during storage period (*Sp*) with the increasing of TS temperature (Figure 1B) and time (Figure 1C). As expected, during storage all TS-treated samples showed an L* value reduction due to fruit ripening. Comparing the observed values for Ctr samples after 15 days of storage (Table 1), and those in the range of thermosonication temperatures and times used in this study (examples: 30 °C during 35 min, 36 °C during 40 min, 42–44 °C during 10 min and 46 °C during 20 min, resulting in L* values of 47.0, 47.3, 50.9 and 51.6, respectively), it can be concluded that TS delays color development and ripening. As a climacteric fruit, tomatoes depend on ethylene for coordinated ripening. Highly intensive treatments can inhibit the ethylene process and consequently many ripening processes, including fruit color changes and aroma development [38]. It is also possible that higher intensive treatments had an adverse effect on the pigment stability of tomatoes due to cavitation, as reported by [39] in anthocyanins.

Few studies reported the effects of thermosonication treatments on product colors: e.g., watermelon juice [22], tomato juice [40], red bell peppers, strawberries, and watercress [41], and shredded carrots [11]. In all these works a decrease of luminosity due to thermosonication has been verified. 

#### 3.2.2. Effect of Thermosonication on Tomato Texture

**Figure 2:**
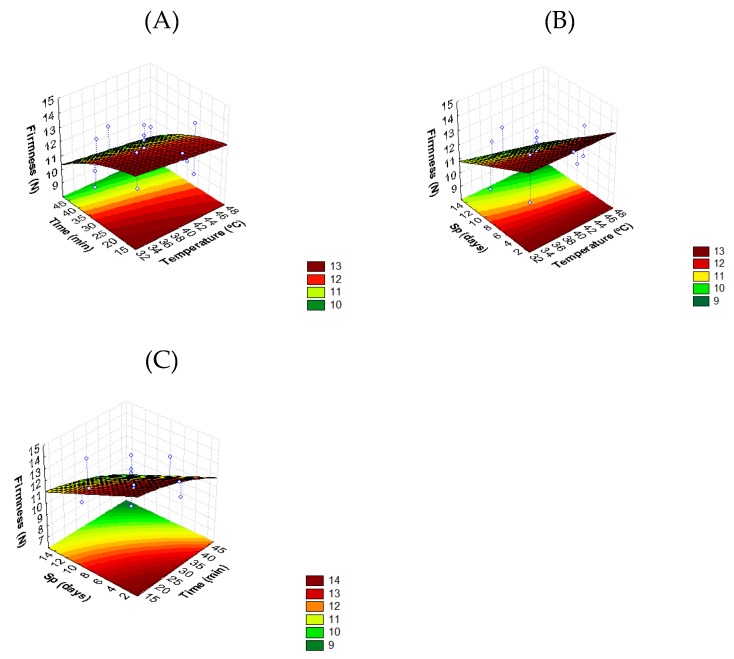
Response surface 3D contour plot indicating the effects of (**A**) temperature (*T*, °C) and time (*t*, min), (**B**) temperature (*T*, °C) and storage period (*Sp*, days), and (**C**) time (*t*, min) and storage period (*Sp*, days) on firmness (maximum force, N) of tomato fruit.

Figure 2 presents the *T*, *t*, and *Sp* effects on tomato firmness. Model coefficients for this parameter (Equation (4)) shows that the interaction of temperature and storage period had the most significant effect on texture with the largest coefficient (−0.006 *T* × *Sp*), followed by treatment time (−0.0013 *t*^2^). It can be seen (Figure 2) that temperature alone did not affect fruit firmness. Treatments between 32 °C and 44 °C at 10–30 min (Figure 2A,B) lead to a slight decrease in fruit firmness after two days of storage (±13.2 N) compared with untreated samples (Table 1). During storage, all TS-samples showed a reduction of firmness when compared to the value measured at day zero (immediately after treatment), however the conditions which best retained the maximum force of the whole tomato along the postharvest period were at 32 °C during 13 min; 35 °C during 20 min; and 40 °C during 30 min, since the firmness at day 15 showed a higher value compared with the value recorded for the untreated sample (Table 1). Regarding the value obtained for untreated samples (9.9 N) after the same storage period, a reduction of 31% of firmness was observed (Table 1). The differential softening observed at different treatment conditions is probably associated with the different ethylene production showed by the untreated and the thermosonicated tomatoes. On the other hand, it seems possible that high power treatments had destructive effects on cell wall constituents, as observed by Aday et al. [42] for strawberries.

In a study developed by Alexandre et al. [41], it was shown that thermosonication at an ultrasound frequency of 35 kHz and power levels of 120 W at 50° and 65 °C during two min better retained the firmness of red bell peppers and strawberries in comparison with heat blanched samples at the same temperature. Alegria et al. [11] revealed that shredded carrots thermosonicated at 50 °C during one min, and ultrasound frequency of 45 kHz and 80% of power level had the best fresh firmness perception (rating 1.7 on a scale from 1 (very firm) to 2 (firm)), compared with an extreme softening heat treatment.

#### 3.2.3. Effect of Thermosonication on Tomato Total Phenolic Content

**Figure 3:**
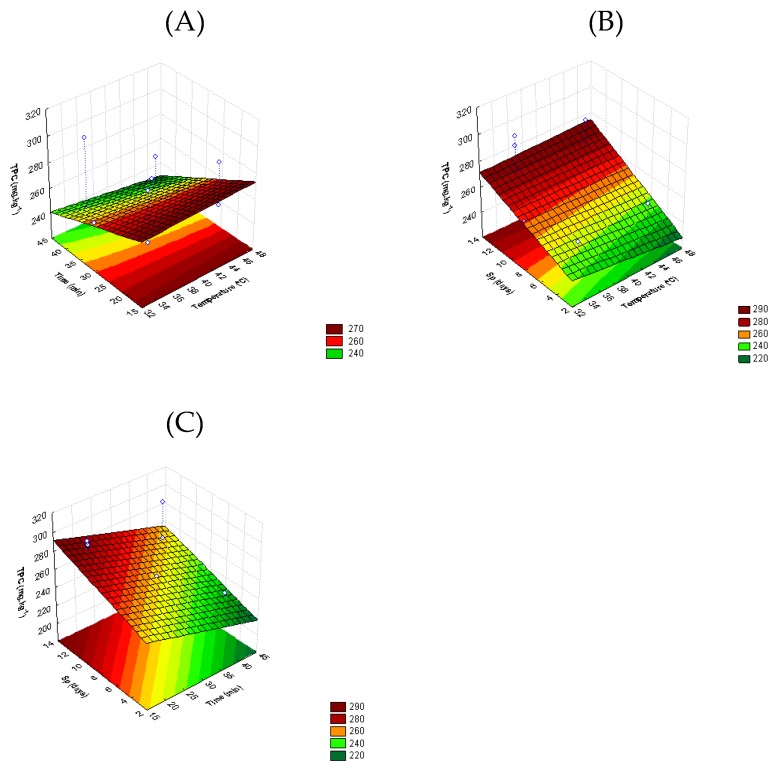
Response surface 3D contour plot indicating the effects of (**A**) temperature (*T*, °C) and time (*t*, min), (**B**) temperature (*T*, °C) and storage period (*Sp*, days), and (**C**) time (*t*, min) and storage period (*Sp*, days) on total phenolic content (TPC, mg Kg^−1^) of tomato fruit.

The interaction of temperature and time (*T* × *t*) and temperature and storage period (*T* × *Sp*) significantly influence (*p* > 0.05) the total phenolic content (TPC) of tomato fruit (Equation (5)) (Figure 3). 

TPC of untreated samples was 243 ± 8 mg.kg^−1^, and immediately after TS treatments an increase of TPC value was obtained for all treatments (Figure 3A). Treatments at higher temperatures (>32 °C) for shorter periods (<30 min) increased fruit TPC (approximately 60 mg GAE.kg^−1^), compared to untreated tomato composition. After day 15, samples treated at 32 °C during 13 min, 35 °C during 20 min, and 40 °C during 30 min reached a TPC increase of 29%, 25%, and 19%, respectively.

In spite of the lack of literature on the effect of thermosonication on TPC of whole tomatoes, Rawson et al. [22] found a decrease in value of TPC in watermelon juice as the temperature was increased from 25 to 45 °C, being more pronounced at higher treatment times. According to Adekunte et al. [40], cavitation induced by ultrasounds has been responsible for various physical and chemical reactions, such as accelerating chemical reactions or increasing diffusion rates, leading to loss of some bioactive compounds. On the contrary, in our study thermosonication led to tomato phenolic compound increase. This effect may be associated to the abiotic stresses that affect the phenylpropanoid pathway responsible for phenolic synthesis, which produce high levels of phenolics as a stress response [43].

#### 3.2.4. Effect of Thermosonication on Tomato Weight Loss

**Figure 4:**
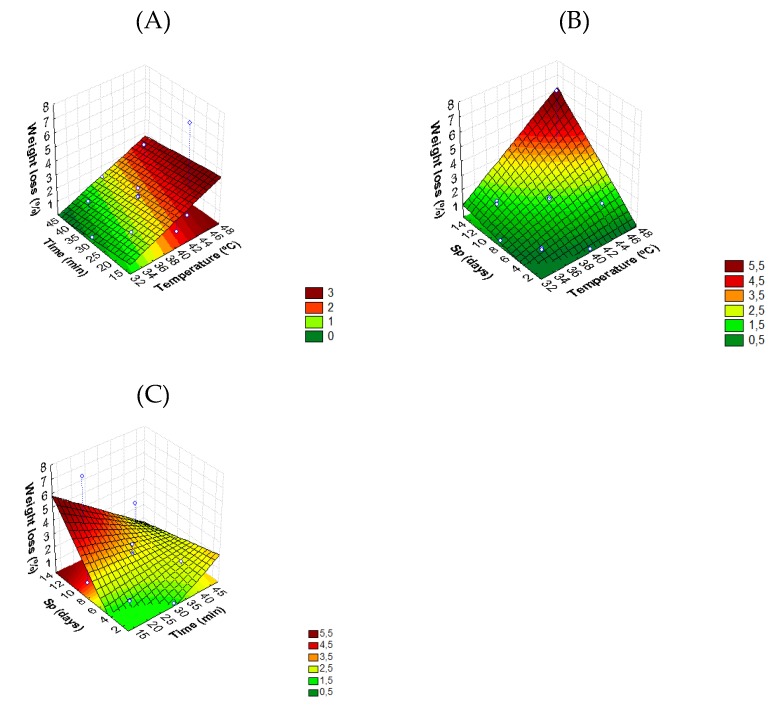
Response surface 3D contour plot indicating the effects of (**A**) temperature (*T*, °C) and time (*t*, min), (**B**) temperature (*T*, °C) and storage period (*Sp*, days), and (**C**) time (*t*, min) and storage period (*Sp*, days) on weight loss (%) of tomato fruit.

Tomato weight loss is linearly dependent on treatment time (*t*) and interactions between temperature and storage period (*T* × *Sp*), and time and storage period (*t* × *Sp*) (Figure 4).

Figure 4C shows that samples with treatment times lower than 30 min do not suffer any weight loss (WL = 0), and total weight is also maintained during the first two days of storage for TS temperatures of 30–34 °C (Figure 4B). Samples treated with higher temperatures (>45 °C) for 15 min presented greater weight losses (4.4%) (Figure 4A). In a previous study developed by Valero et al. [44], the weight loss was denoted as an important and determinant attribute for fruit quality. During the postharvest of tomato fruit, water loss is achieved, leading to a decline of quality [45]. Most commodities become unsalable as fresh produce after losing 3–10% of their weight [46]. Our study revealed that thermosonication treatment in a temperature range of 32–40 °C for time periods of 13–47 min maintains a lower weight loss of fresh tomatoes. This beneficial effect indicates that thermosonication at these conditions can be effective in retaining the moisture of the tomato. No information in the scientific literature has been found regarding this action on TS-treated tomatoes. Moreover, the combination of ultrasounds and heat treatment allowed for maintenance in fruit appearance and was better accepted by the sensory panel, as referenced by Pinheiro et al. [33].

Nevertheless, the WL model (Equation (6)) presented a significant (*p* < 0.05) lack-of-fit, suggesting that the source of variance is due to the inadequacy of the polynomial model to reproduce experimental data.

### 3.3. Thermosonication Treatment Condition Optimization and Validation

The thermosonication conditions for tomato fruit can be considered optimum if fruit decay incidence is minimum and quality parameters, like color, texture, and TPC, attain minimal changes along storage. 

According to previous evaluation of all models, three thermosonication conditions with lower, medium, and higher treatment intensity (32 °C during 13 min, 35 °C during 20 min, and 40 °C during 30 min) were selected in order to validate predicted results. Under these treatment conditions a delay of color development and minimal firmness changes, as well as an increase of phenolic compounds, were observed during storage.

In order to confirm the effects of previously optimized TS conditions on color, texture, TPC, and sensory analysis on whole tomato fruit, a comparison experiment was performed on fruit stored at 10 °C for 15 days. 

**Figure 5:**
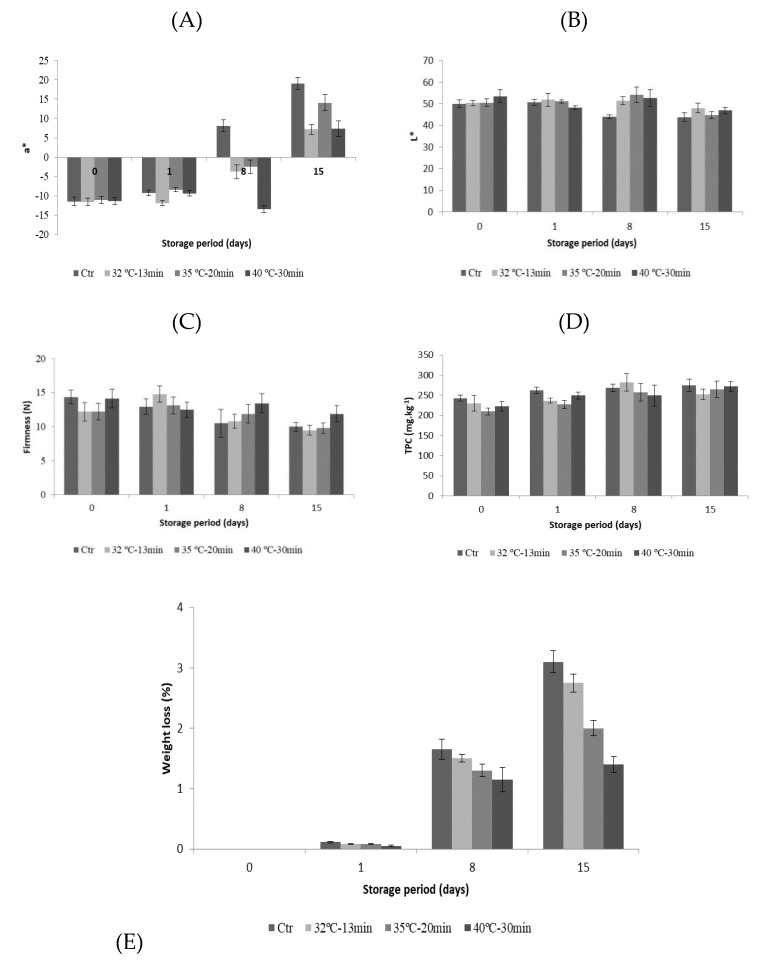
Evaluation of a* (**A**) and L* (**B**) color parameter, firmness (**C**), total phenolic content (**D**), and weight loss (**E**) of untreated (Ctr) and TS-treated tomato fruit during 15 days at 10 °C. Vertical bars represent standard deviation.

The major pigments of tomato color are a mix of red and yellow pigments, mainly due to the presence of lycopene, β-carotenoid, and lutein [47]. According to Abushita et al. [48], 75–83% of the red color developed during ripening of tomatoes is due to lycopene. Thus, a* value should also be considered a good physical parameter to describe the visual color degradation. Figure 5A,B present a* and L* color evaluation during storage at 10 °C.

Immediately after thermosonication, no significant changes (*p* > 0.05) were observed on a* value of TS-treated samples compared to untreated fruit, since a variation of only 0.33 units of a* was observed. The rate of red color development of TS-treated samples at 32 °C during 13 min and 40 °C during 30 min showed identical behavior during storage, 1.3 and 1.2 a* units/day, respectively, while Ctr samples present the highest rate of 3.0 units/day. After thermosonication, only the TS-treated sample at 40 °C during 30 min showed a significant increase (*p* < 0.05) of luminosity (6%) compared to Ctr samples. At the end of storage, a decrease on L* value was observed in all samples, having the TS-treated samples at 32 °C during 13 min and 40 °C during 30 min as the highest values, representing a delay in red color development and ripening.

Firmness of untreated tomatoes was 14.4 ± 1.0 N, and after thermosonication a reduction on all TS-treated samples was denoted, ca. 15% for both samples at 32 °C during 13 min and 35 °C during 20 min, and only 2% when the conditions of 40 °C during 30 min were applied (Figure 5C). At the end of storage, a decrease of this parameter in all samples was observed with the increase of thermosonication intensity: 30%, 22%, 20% and 15% for Ctr, 32 °C during 13 min, 35 °C during 20 min, and 40 °C during 30 min, respectively. 

After the thermosonication treatments, a reduction on tomato TPC was observed, with the conditions of 32 °C during 13 min and 40 °C during 30 min conducting the lower reduction on this parameter, 5% and 8%, respectively, compared with Ctr samples (243 mg kg^−1^) (Figure 5D). During storage an increase of TPC was observed, whereas the TS-treated samples at 35 °C during 20 min and 40 °C during 30 min reveal the highest content at the end of storage: 26% and 22%, respectively. The weight loss (WL) of all samples increased during storage at 10 °C (Figure 5E). After day 15, the samples treated with TS showed the lowest WL compared to Ctr samples, whereas the 40 °C during 30 min denoted only a loss of 1.4%.

The effects of the optimized thermosonication conditions on sensory attributes, color, global acceptability, and deterioration index are presented in Table 5. In terms of acceptability and the deterioration index, all samples showed differences from day eight, being more pronounced on Ctr samples and reaching to between moderately and medium acceptable, which is defined as the consumer limit. Comparing the TS-treated samples, at 32 °C during 13 min less decay was observed; however, no significant differences were detected between treated samples. The sensorial color evaluation for TS-treated samples are similar to the untreated sample (Ctr). Untreated and TS-treated samples at 35 °C during 20 min denoted a rapid change of visual color from day eight, both with registering scores of 5.8, while the other samples ranged from 3.7 to 3.8. During storage, color of Ctr samples was evaluated to score 6.0 (>90% red), however, all the TS-treated fruit reached this value after the same period (15 days of storage), with exception of samples treated at 40 °C during 30 min, revealing a delay of red development. These results demonstrate that thermosonication at 40 °C during 30 min was optimum in terms of reducing fruit decay and maintaining tomato quality during refrigerated storage.

Color rating system: 1 = green (0% red); 2 = breaker (<10% red); 3 = turning (10% < red < 30%); 4 = pink (30% < red < 60%); 5 = red (60% < red < 90%) and 6 = red (>90% red).

Global acceptability rating system: 1 = highly acceptable; 2 = moderately acceptable; 3 = medium acceptable (consumer limit); 4 = moderately unacceptable; 5 = unacceptable. 

Deterioration index rating system: 0 = absent; 1 = very slight; 2 = moderate; 3 = severe.

## 4. Conclusions

This study demonstrated that the second-order polynomials are an effective technique for investigating color, firmness, and total phenolic content degradation as a function of temperature (°C) and time (min) of TS treatment and refrigerated storage period (days) for fresh mature-green tomato cv. Zinac samples. Thermosonication treatment has potential for delaying ripening changes with consequent maintenance of tomato fruit quality. It can be concluded that optimal thermosonication conditions for tomato fruit should be at 40 °C during 30 min at an ultrasound frequency of 45 kHz and power level of 80%. In the light of these findings, the potential for optimization of the thermosonication treatments in the studied postharvest method can be a promising alternative to conventional tomato treatments for controlling physicochemical changes, microbial load development, and nutritional degradation during refrigerated storage.

## Figures and Tables

**Table 1:** Quality attributes of untreated tomatoes (Ctr samples) stored at 10 °C during 0 and 15 days (average ± standard deviation).

Quality Attributes	Storage Days	
	0	15
Color parameters		
L*	50.1 ± 1.7	43.8 ± 2.0
a*	−11.5 ± 1.2	19.1 ± 1.6
b*	21.5 ± 2.2	26.8 ± 1.6
* °h*	118.1 ± 1.8	54.4 ± 2.7
Texture		
Firmness (N)	14.4 ± 1.0	9.9 ± 0.6
Total phenolic content		
TPC (mg.kg^−1^)	242.8 ± 7.7	274.1 ± 15.5
Weight loss		
WL (%)	0.0 ± 0.0	3.1 ± 0.1

L*—Luminosity (0(black) to 100(white)); a*—Greenness to redness (−60 to +60); b*—Blueness to yellowness (−60 to +60); *°h* —Tonality (0° to 360°).

**Table 2:** Coded and decoded matrix of independent variables.

Coded Independent Variables			Decoded Independent Variables		
X_1_	X_2_	X_3_	Temperature (°C) (*T*)	Time (min) (*t*)	Storage Period (days) (*Sp*)
−1	−1	−1	35	20	4
0	0	0	40	30	8
0	0	0	40	30	8
−1.68179	0	0	32	30	8
1	−1	1	45	20	12
1.68179	0	0	48	30	8
1	1	−1	45	40	4
0	1.68179	0	40	47	8
−1	−1	1	35	20	12
1	−1	−1	45	20	4
0	0	−1.68179	40	30	1
1	1	1	45	40	12
0	−1.68179	0	40	13	8
−1	1	1	35	40	12
−1	1	−1	35	40	4
0	0	1.68179	40	30	15

**Table 3:** Analysis of variance of the second order polynomial model for color parameter (L*), firmness, total phenolic content (TPC), and weight loss (WL) of thermosonicated tomatoes.

Effect	Source	SS	d*f*	MS	F-ratio (Model Significance)	*p*
L*	Regression	74.11	5	14.82	7.57 ^a^	0.0035
	Residual	19.58	10	1.96		
	Lack-of-fit	19.43	9	2.16	13.80 ^b^	0.21
	Pure Error	0.16	1	0.16		
	Total	93.7	15			
Firmness	Regression	22.62	2	1.31	6.72 ^a^	0.01
	Residual	21.87	13	1.68		
	Lack-of-fit	21.81	12	1.82	32.63 ^b^	0.14
	Pure Error	0.056	1	0.056		
	Total	44.49	15			
TPC	Regression	5897.98	2	2948.99	268.25	0.04
	Residual	3761.23	13	289.32		
	Lack-of-fit	3750.21	12	312.52	28.43	0.15
	Pure Error	10.99	1	10.99		
	Total	9659.21	15			
WL	Regression	33.05	3	11.02	13.21 ^a^	0.000
	Residual	10.01	12	0.83		
	Lack-of-fit	10.01	11	0.91	406.71 ^b^	0.04
	Pure Error	0.002	1	0.002		
	Total	43.06	15			

SS—Sums of squares; d*f*—degrees of freedom; Ms—Mean square; ^a^ F (MS_regression_/MS_residuals_); ^b^ F (MS_lack-of-fit_/MS_Pure error_); *F* test significant at *p* < 0.05.

**Table 4:** Verification of predictive model Equations (3)–(5), respectively, for L* value, firmness, and TPC in whole tomatoes by thermosonication during storage at 10 °C.

Run	Variables			L*		Firmness		TPC	
	*T* (°C)	*t* (min)	*Sp* (days)	Predicted	Observed ^a^	Predicted	Observed ^a^	Predicted	Observed ^a^
1	30	20	2	53.4	49.8 ± 0.6	14.0	13.4 ± 0.4	251.7	245.1 ± 1.9
2	33	18	6	53.5	52.2 ± 0.4	13.3	12.5 ± 0.3	259.9	257.0 ± 3.3
3	35	15	10	52.2	50.4 ± 0.3	12.5	11.9 ± 0.1	278.0	283.4 ± 6.9
4	37	25	5	52.0	50.4 ± 0.4	13.0	11.6 ± 0.1	247.9	235.5 ± 4.8
5	38	7	8	52.8	51.7 ± 0.3	13.0	11.7 ± 0.3	276.2	275.2 ± 4.6
6	42	16	15	46.1	45.6 ± 0.4	10.8	9.4 ± 0.4	305.4	300.1 ± 2.0
7	44	22	22	39.1	34.8 ± 0.2	8.4	7.8 ± 0.2	362.4	304.8 ± 3.3
8	46	15	16	43.0	43.6 ± 0.3	10.2	9.6 ± 0.2	312.7	304.6 ± 4.9
9	47	5	5	46.2	44.7 ± 0.3	13.4	12.5 ± 0.2	267.2	256.6 ± 2.3
10	49	3	8	42.2	42.5 ± 0.6	12.5	10.6 ± 0.2	279.5	275.9 ± 4.6

^a^ Values represent average ± standard deviations (*n* = 3).

**Table 5:** Effect of optimized thermosonication treatments on sensory attributes of stored whole tomato fruits (color, global acceptability, and deterioration index).

Sensory Analysis	Storage Period (days)	Ctr	32 °C-13 min	35 °C-20 min	40 °C-30 min
**Color**	0	1.0 ^a^ ± 0.0	1.0 ^a^ ± 0.0	1.0 ^a^ ± 0.0	1.0 ^a^ ± 0.0
	1	2.8 ^c^ ± 0.3	1.0 ^a^ ± 0.3	1.0 ^a^ ± 0.3	1.0 ^a^ ± 0.3
	8	5.8 ^f^ ± 0.3	3.8 ^e^ ± 0.3	5.8 ^f^ ± 0.3	3.7 ^e^ ± 0.3
	15	6.0 ^f^ ± 0.0	6.0 ^f^ ± 0.0	6.0 ^f^ ± 0.0	5.8 ^f^ ± 0.3
**Global acceptability**	0	1.0 ^a^ ± 0.0	1.0 ^a^ ± 0.0	1.0 ^a^ ± 0.0	1.0 ^a^ ± 0.0
	1	1.0 ^a^ ± 0.0	1.0 ^a^ ± 0.0	1.0 ^a^ ± 0.0	1.0 ^a^ ± 0.0
	8	2.1 ^bc^ ± 0.2	1.0 ^a^ ± 0.2	2.0 ^bc^ ± 0.2	2.0 ^b^ ± 0.2
	15	2.5 ^d^ ± 0.1	2.1 ^bc^ ± 0.2	2.3 ^cd^ ± 0.2	2.1 ^bc^ ± 0.1
**Deterioration index**	0	0.0 ^a^ ± 0.0	0.0 ^a^ ± 0.0	0.0 ^a^ ± 0.0	0.0 ^a^ ± 0.0
	1	0.0 ^a^ ± 0.0	0.0 ^a^ ± 0.0	0.0 ^a^ ± 0.0	0.0 ^a^ ± 0.0
	8	0.6 ^d^ ± 0.1	0.5 ^cd^ ± 0.1	0.58 ^bc^ ± 0.0	0.3 ^b^ ± 0.1
	15	1.5 ^g^ ± 0.1	1.3 ^f^ ± 0.1	1.2 ^f^ ± 0.1	0.9 ^e^ ± 0.1

Values are an average of 8–10 observations. In the same line, different letters represent significant differences at *p* < 0.05.
